# Fibrillar and Micellar Aggregation of Semaglutide and Formation of a Chiral-Imprinted Glass

**DOI:** 10.1021/acs.biomac.5c02669

**Published:** 2026-03-31

**Authors:** Valeria Castelletto, Lucas R. de Mello, Jani Seitsonen, Ian W. Hamley

**Affiliations:** † School of Chemistry, Food Biosciences and Pharmacy, 6816 University of Reading, Whiteknights, Reading, Berkshire RG6 6AD, U.K.; ‡ Nanomicroscopy Center, 174277 Aalto University, Puumiehenkuja 2, FIN-02150 Espoo, Finland

## Abstract

Semaglutide is a therapeutically important lipopeptide that comprises a lipidated peptide with a glucagon-like peptide-1 (GLP-1) sequence, and may be prone to aggregation. We show that semaglutide in low pH 2.4 solutions forms β-sheet fibrils, in contrast to the oligomeric and micellar structures formed at higher pH. Based on cryo-TEM images showing twisted fibrils and the modeling of SAXS data (and with knowledge from fiber XRD) and molecular dynamics simulations, a model for the β-sheet structure is proposed, which comprises curved β-strands arranged in an antiparallel fashion around a core that comprises the lipidated lysine residue. This structure results from the patterning of the charged, polar, hydrophobic, and lipidated residues. Remarkably, it is possible to form a glass from the base form of semaglutide with crotonic acid, an organic salt capable of hydrogen bonding. Semaglutide glasses may have applications in biomedicine or therapeutics (for example, as slow-release depots).

## Graphical abstract

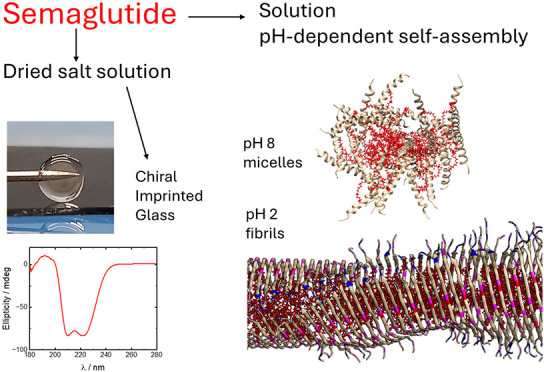

## Introduction

The treatment of obesity, diabetes, and disease or ill health related to or exacerbated by these conditions is a major global healthcare challenge. Food intake, satiety, and gastric emptying are regulated by many gut hormones, including glucagon-like peptide-1 (GLP-1), which regulates the production of insulin and glucagon.^1–5^ GLP-1 receptor agonists represent an important new class of therapeutics and, to date, are represented by lipidated peptides directly derived from the GLP-1 sequence^6–11^ or peptides with sequence homology to GLP-1, such as exenatide.^1^ GLP-1-based lipopeptides, including semaglutide and tirzepatide, are now established as effective treatments for diabetes and obesity and are being investigated as therapeutics for a range of other conditions.^6–11^ Injectable formulations of semaglutide are marketed as Ozempic for diabetes or Wegovy for weight loss. Tirzepatide is known as Mounjaro for the treatment of diabetes or Zepbound for obesity. These molecules were designed starting from the native GLP-1 peptide sequence with one or more substitutions with non-natural amino acids (and other sequence modifications) to improve stability and reduce enzymatic cleavage in vivo. The structure of Semaglutide is shown in Scheme 1, and it contains 31 residues and a lipid chain conjugation on Lys-20 involving a C_18_ carboxylic acid chain attached via an ethylene glycol-based spacer. The attachment of lipid chains enhances serum albumin binding to provide better stability in vivo, allowing once-weekly administration.^6,7^

**Scheme 1:**
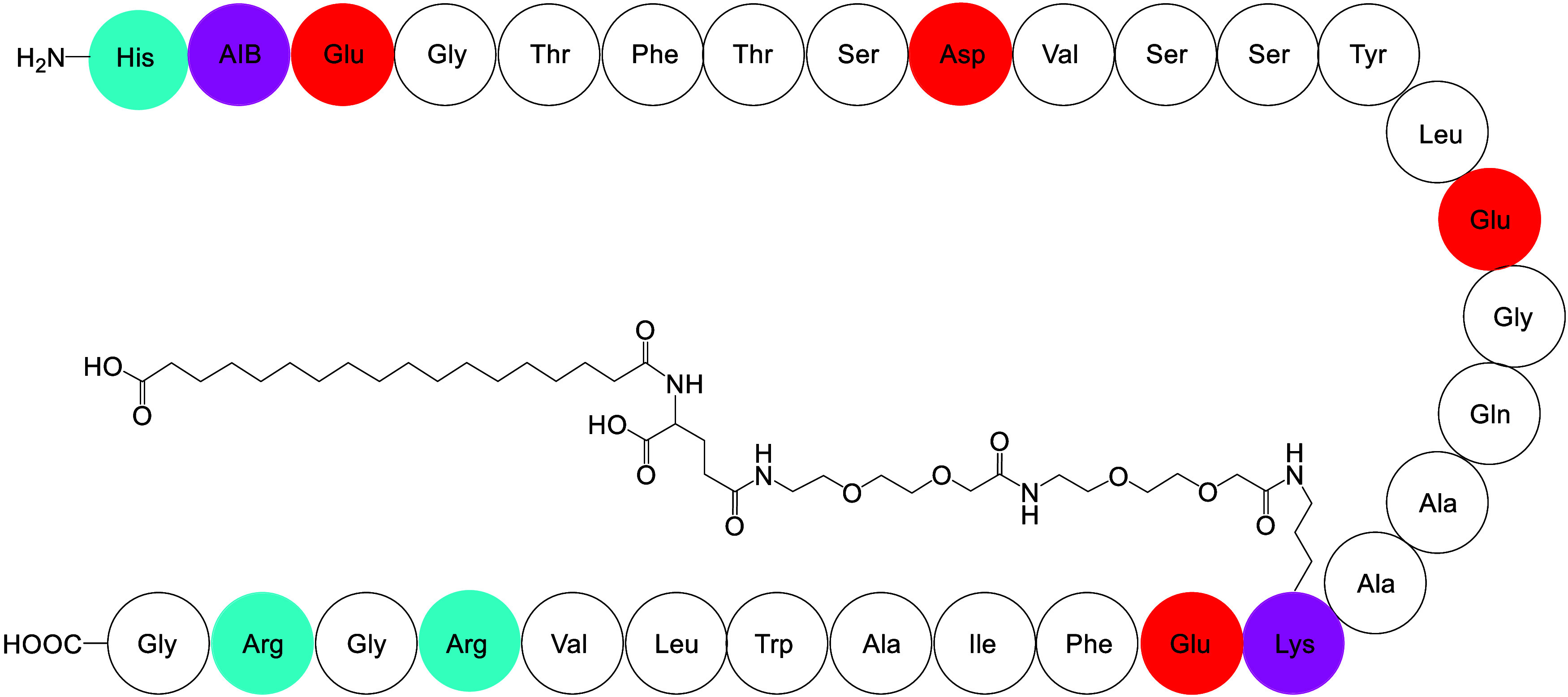
Molecular Structures of Semaglutide^a^

a Non-natural residues colored mauve (AIB = 2-aminoisobutyric acid), anionic red, and cationic cyan. A side chain attached to Lys-20 molecules comprises a diEG_2_-γ-Glu linker (EG: ethylene glycol) and a C_18_ (octadecadienoic) carboxylic acid.

Semaglutide is a lipopeptide (a type of peptide amphiphile), and the lipid chain conjugated to the peptide may impart a propensity for the molecule to aggregate through self-assembly due to the amphiphilicity of the molecule. Many types of lipopeptides are now known to self-assemble into a wide variety of nanostructures depending on sequence, lipid chain length and position, solution conditions, and other variables.^12–15^ Research to date has primarily focused on N-terminally lipidated peptides containing bioderived or bioinspired synthetic sequences. The aggregation properties of lipidated gut hormone peptides have also been examined.^16–22^ It has been shown that human GLP-1 aggregates into β-sheet fibrils, and the aggregation kinetics of this process were studied using the fluorescent probe Thioflavin T (ThT).^17^ Further studies have probed the structure of off-pathway low molecular-weight oligomers of GLP-1 (and the C-terminal amidated analogue).^23^ Lipidation is found to influence GLP-1 aggregation and reduces solubility (depending on pH and leads to oligomers that are larger and more stable than those for the parent GLP-1 peptide.^22^ Several aggregate structures were shown for the lipidated analogues (including the C-terminal amidated liraglutide-Am and semaglutide-Am) studied.^22^ Other work suggests that liraglutide (another GLP-1-based lipopeptide) forms small micelle-like oligomers with a pH-dependent change in micelle structure.^16^ The aggregation of liraglutide demonstrates a memory effect, depending on the oligomerization state of the lyophilized solution.^24^ The oligomerization state can also influence subsequent fibril formation.^24^ Various physicochemical factors, including peptide sequence, charge distribution, pH, and concentration, as well as formulation considerations, that influence the stability and aggregation behavior of peptide-based therapeutics have been discussed.^18^

There is great interest in the applications of semaglutide and its properties, although there are few studies to date on its aggregation. A semaglutide derivative has been reported to form aggregates above a critical concentration in aqueous buffer solution.^25^ Time-resolved tryptophan fluorescence and fluorescence anisotropy measurements provided information on the size of the aggregates, and this was complemented by molecular dynamics (MD) simulations, which point to the initial formation of dimers followed by the formation of larger aggregates over a longer period (40 days).^25^ The slow (period of weeks) emulsion formation of semaglutide has been observed in the presence of hydrophobic surfaces.^26^ SAXS and dynamic light scattering were used to probe the structures of the microemulsions. At pH 7.5, semaglutide-Am (with amidated C-terminus) has been reported to have an α-helical CD spectrum, and on the basis of fluorescence probe experiment using ThT or ANS (8-anilino-1-naphthalenesulfonic acid), no aggregation could be detected after 6 days under these conditions (although oligomers were detected in fresh solutions).^22^ However, in contrast, we recently showed using small-angle X-ray scattering (SAXS), cryo-TEM imaging, and MD simulations that semaglutide (uncapped, base form) in dilute solution at pH 8 forms dimeric or trimeric aggregates, whereas at sufficiently high concentration micelles were observed, after aging.^27^ The micelles have a radius of 2.3 nm and an estimated association number around *p* = 30. At lower concentrations, smaller dimeric and oligomeric structures are present. Circular dichroism (CD) spectroscopy showed that the molecule has an α-helical conformation under these conditions.

Here, we first investigate the self-assembly of semaglutide in water, comparing aggregation under neutral and acidic pH conditions. We use cryogenic-TEM together with SAXS and MD simulations to elucidate the aggregate structures, along with spectroscopic methods to probe peptide conformation and to determine the critical aggregation concentration (CAC). Unexpectedly, we find that semaglutide forms well-defined narrow fibrils at pH 2 with a β-sheet structure. This behavior is in marked contrast to the α-helical dimers/trimers (low concentration) or micelles (high concentration) that form at pH 8.^27^ The structure of the fibrils is modeled via atomistic molecular dynamics (MD simulations) with constraints from cryo-TEM, SAXS, and fiber XRD. This leads to a proposed structure comprising curved antiparallel β-sheets arranged pairwise around a fiber core comprising the lipid chains o the lysine residue (Scheme 1). In addition, we found that the slow evaporation of an aqueous solution of semaglutide with crotonic acid leads to glass formation. The glass shows high transparency and an accessible glass transition temperature, as well as fluorescence. Most notably, the α-helical peptide structure is retained in the vitrified sample. SAXS/WAXS also suggests that the glass is formed by vitrification of an initial oligomer/micellar solution, thus representing a chiral-imprinted glass.

## Experimental Section

### Materials and Sample Preparation

Semaglutide (TFA) was purchased from Peptide Synthetics (Peptide Protein Research Ltd., Farnham, UK). The molar mass measured by ESI-MS is 4113.9 g mol^–1^ (4113.6 g mol^–1^ expected). Purity (by HPLC) is 99.4%.

Semaglutide (base form) was purchased from Bioserv (Calibre Scientific, Rotherham, UK). The purity by HPLC was >95%, and it was supplied in nonsalt form. The molar mass is *M* = 4117.2 g mol^–1^ (4113.6 g mol^–1^expected). Purity (by HPLC) is 97.8%.

Characterization data are presented in Figures S1 and S2 for the two forms of semaglutide. The purity and characteristic mass spectra are similar for both forms of semaglutide studied. The pH of semaglutide (base) is pH 8 for a 1 wt % aged sample (pH 7 reported previously^27^), and the value for semaglutide (TFA) was found to be pH 2.4.

Solutions were prepared using weighed amounts of semaglutide salts in ultrapure water. To monitor the aggregation and stability, some samples were left to age up to 40 days in the fridge at 4 °C, protected from the light. For pH adjustment studies, 1 M HCl solution was added to a sample of semaglutide (base) with native pH 8.

Glasses were prepared using Semaglutide (base), by dissolving the semaglutide in a solution of 0.21 wt % crotonic acid at a 1:1 or 1:1.3 [semaglutide]:[crotonic acid] molar concentration ratio. The final solution contained 10 or 8 wt % semaglutide dissolved in a solution of 0.21 wt % crotonic acid. For the 8 wt % precursor solution, we measured pH 7, and for the 10 wt %, it was pH 9. The final solution was homogenized by 20 min of alternated ultrasound and vigorous vortex cycles. Thereafter, a 20 μL drop of the final solution was placed on a silicone rubber tape and left to dry for 24 h inside a sealed desiccator loaded with silica gel. Following this procedure, the drop of the final solution turned into a flat disc of glass, which was easily detached from the surface of the silicon rubber tape.

#### Circular Dichroism (CD) and UV/Vis Spectroscopy

Far-UV CD spectra were collected by using a Chirascan spectropolarimeter (Applied Photophysics, Leatherhead, UK) equipped with a thermal controller. Spectra were recorded from 180 to 400 nm. For solutions, samples were mounted in a quartz cell with detachable windows with 0.01 or 0.1 mm path length. The CD spectra from the samples were corrected by water background subtraction and were smoothed using the Chirascan Software. The residue of the calculation was chosen to oscillate around the average to avoid artifacts in the smoothed curve. CD data, measured in mdeg, was normalized to molar ellipticity using the molar concentration of the sample and the cell path length. For the glass, a precursor aqueous solution of 8.4 wt % semaglutide (base form, pH 8) with 0.21 wt % crotonic acid was dried between 0.01 mm parallel plaques. The CD and UV/vis absorption spectra were measured in parallel on the Chirascan instrument.

#### FTIR Spectroscopy

FTIR spectra were obtained by using a Thermo-Scientific Nicolet iS5 instrument with a DTGS detector. The solution was placed in a Specac Pearl liquid cell with CaF_2_ plates. For each sample, a total of 128 scans were recorded over the range of 900–4000 cm^–1^, with a resolution of 2 cm^–1^.

#### Fluorescence Spectroscopy

Fluorescence emission spectra were measured by using a Varian Model Cary Eclipse spectrofluorometer. Solutions were loaded in a 10 mm light path quartz cell. The solutions were excited at 280 nm, and the emission fluorescence was measured from 300 to 500 nm. The wavelength of excitation was chosen from the corresponding peak of the absorption measured in the UV–vis measurement. The fluorescence spectra for the glasses were measured with the same cells as for the CD experiments (same sample details, dried between 0.01 mm quartz plaques), and the excitation wavelength was λ = 280 nm. To determine the Critical Aggregation Concentration (CAC), fluorescence spectroscopy assays were performed using the fluorescent probe Thioflavin T (ThT) used to identify the amyloid β-sheet structure. Samples were placed in 4 mm inner-width quartz cuvettes. ThT fluorescence assays were conducted using a series of semaglutide solutions dissolved in 5 × 10^–3^ wt % ThT. The spectra were recorded from 460 to 600 nm using an excitation wavelength of λ_ex_ = 440 nm.

#### Cryogenic-TEM (Cryo-TEM)

Imaging was carried out using a field emission cryo-electron microscope (JEOL JEM-3200FSC), operating at 200 kV. Images were taken in bright field mode and using zero-loss energy filtering (omega type) with a slit width of 20 eV. Micrographs were recorded using a Gatan Ultrascan 4000 CCD camera. The specimen temperature was maintained at −187 °C during the imaging. Vitrified specimens were prepared using an automated FEI Vitrobot device using Quantifoil 3.5/1 holey carbon copper grids with a hole size of 3.5 μm. Just prior to use, grids were plasma cleaned using a Gatan Solarus 9500 plasma cleaner and then transferred into the environmental chamber of a FEI Vitrobot at room temperature and 100% humidity. Thereafter, 3 μL of sample solution was applied on the grid, and it was blotted twice for 5 s and then vitrified in a 1/1 mixture of liquid ethane and propane at a temperature of −180 °C. The grids with vitrified sample solution were maintained at liquid nitrogen temperature and then cryo-transferred to the microscope.

#### Small-Angle X-ray Scattering (SAXS) and Wide-Angle X-ray Scattering (WAXS)

SAXS experiments were performed on beamline B21 ^28^ at Diamond Light Source (Harwell, UK). The sample solutions were loaded into the 96-well plate of an EMBL BioSAXS robot and then injected via an automated sample exchanger into a quartz capillary (1.8 mm internal diameter) in the X-ray beam. The quartz capillary was enclosed in a vacuum chamber to avoid parasitic scattering. After the sample was injected into the capillary and reached the X-ray beam, the flow was stopped during SAXS data acquisition. Beamline B21 operates with a fixed camera length (3.9 m) and a fixed energy (12.4 keV). The images were captured by using a PILATUS 2 M detector. Data were put on an absolute scale with respect to the known absolute scattering intensity of water, and processing was performed using dedicated beamline software ScÅtter. Additional SAXS/WAXS measurements were performed on dried peptide stalk and glass samples mounted in custom-built polycarbonate multipurpose sample holders,^29^ held with Superio (Mitsubishi Chemical) UT F-type poly­(ether imide) film (7 μm thickness) (which provides a very low SAXS background), which were inserted into the sample chamber in the beamline. The WAXS data was acquired using a Dectris Eiger 2 1 M detector, and the q-axis was calibrated using the diffraction spectrum of silver behenate.

#### Fiber XRD

Measurements were performed on the same peptide stalks used for the WAXS experiments. Each stalk was mounted onto a four-axis goniometer of an Oxford Diffraction Gemini Ultra instrument. The sample–detector distance was 60 or 140 mm. The X-ray wavelength λ = 1.54 Å was used to calculate the scattering vector *q* = 4π sin θ/λ (2θ: scattering angle). The detector was a Sapphire CCD.

#### Molecular Dynamics Simulations

Molecular dynamics simulations were performed using Gromacs^30^ (versions 2023.2 and 2023.3-Ubuntu-2023.3). Semaglutide molecules were built with the sequence in an antiparallel β-sheet conformation. Then, antiparallel β-sheet arrays comprising 64 β-strands were built, taking care to retain amide/hydroxyl hydrogen bonds, and these were arranged in an opposed fashion in a model containing 128 β-strands. The lipopeptide structures were generated using UCSF Chimera. Simulations were performed using the CHARMM36 force field^31,32^ with manual patching of force field parameters for the Lys-20 side chain based on parameters for the side chain treated as a ‘ligand’ using CHARMM-GUI.^33,34^ The ionizable residues have the following charges from the N- to C-terminus: N-terminal His +2, Glu −1, Glu −1, Lyc 0, Glu −1, Arg +1, Arg +1, and C-terminus −1.

The fibrils were placed into simulation boxes (cubes) of length 30 nm, and systems were solvated using spc216 water. Each system was neutralized using a matching number of Na^+^ counterions. After energy minimization and 100 ps relaxation stages in the NVT ensemble, the final simulations were carried out in the NPT ensemble in triplicate using a leapfrog integrator with steps of 2 fs up to 10,000 ps (10 ns). The temperature was maintained at 300 K using the velocity-rescale (modified Berendsen) thermostat^35^ with a coupling constant of 10 steps. The pressure was maintained at 1 bar using the Parrinello–Rahman barostat,^36^ and periodic boundary conditions were applied in all three dimensions. The Particle Mesh Ewald scheme^37,38^ was used for long-range electrostatics. Bonds were constrained using the LINCS algorithm,^39^ and the Verlet cutoff scheme^40^ was used. Coulomb and van der Waals cutoffs were 1.0 nm.

#### Scanning Electron Microscopy (SEM)

Glass disks were placed on a stub covered with a carbon tab (Agar Scientific, U.K.) and then coated with gold. A FEI Quanta FEG 600 environmental scanning electron microscope (SEM) in high vacuum mode (5 kV high tension) was used to study and record the SEM images.

#### Differential Scanning Calorimetry (DSC)

Experiments were performed using a TA Instruments Multi-Sample X3 DSC instrument. For the experiments, semaglutide glass was loaded into a TA Instruments Tzero hermetic pan. A ramp rate of 10 °C/min was used for all experiments. The temperature was first decreased from 40 °C to −40 °C. The sample was left to equilibrate at −40 °C for 10 min. A T-ramp −40 °C →120 °C was started following equilibration at −40 °C. This was followed by a final cooling ramp of 120 °C → −40 °C.

## Results and Discussion

The SAXS data shown in Figure 1a provide clear evidence for a dramatic difference in the self-assembly behavior of semaglutide at pH 2.4 and pH 8. As discussed in our previous paper,^27^ at pH 8, semaglutide (base) forms dimers/trimers at low concentration and micelles at a sufficiently high concentration, above a critical micelle concentration (CMC), and the SAXS data comprises a structure factor peak centered at *q* = 0.05 Å^–1^ and form factor features at higher *q*. This remeasured data is quantitatively consistent with our previously reported SAXS results.^27^ In contrast to the micellar structure at pH 8, the SAXS intensity profile for semaglutide (TFA) at pH 2.4 shows a low wavenumber *q* scaling of intensity, *I* ∼ *q* ^–1^, indicating cylindrical fibril structures (Figure 1b). The data can be very well described using a core–shell cylinder form factor. The fit parameters are listed in Table S1. The core cylinder radius *R*_c_ is 20 Å (with considerable polydispersity), consistent with an extended C_18_ chain. The fit also provides a shell thickness of 27 Å (the fibril length *L* in the fits is not uniquely determined in the measured *q* range; since *L* ≫ *R*_c_, it serves as a scaling parameter). The cryo-TEM image in Figure 1c clearly confirms that semaglutide forms fibrils at pH 2.4. The fibrils have an internal twisted structure (as highlighted in an enlarged image shown in Figure 1d) and are quite narrow, consistent with the average radius obtained from SAXS. Additional cryo-TEM images showing extensive fibril structures are provided in Figure S3.

**Figure 1:**
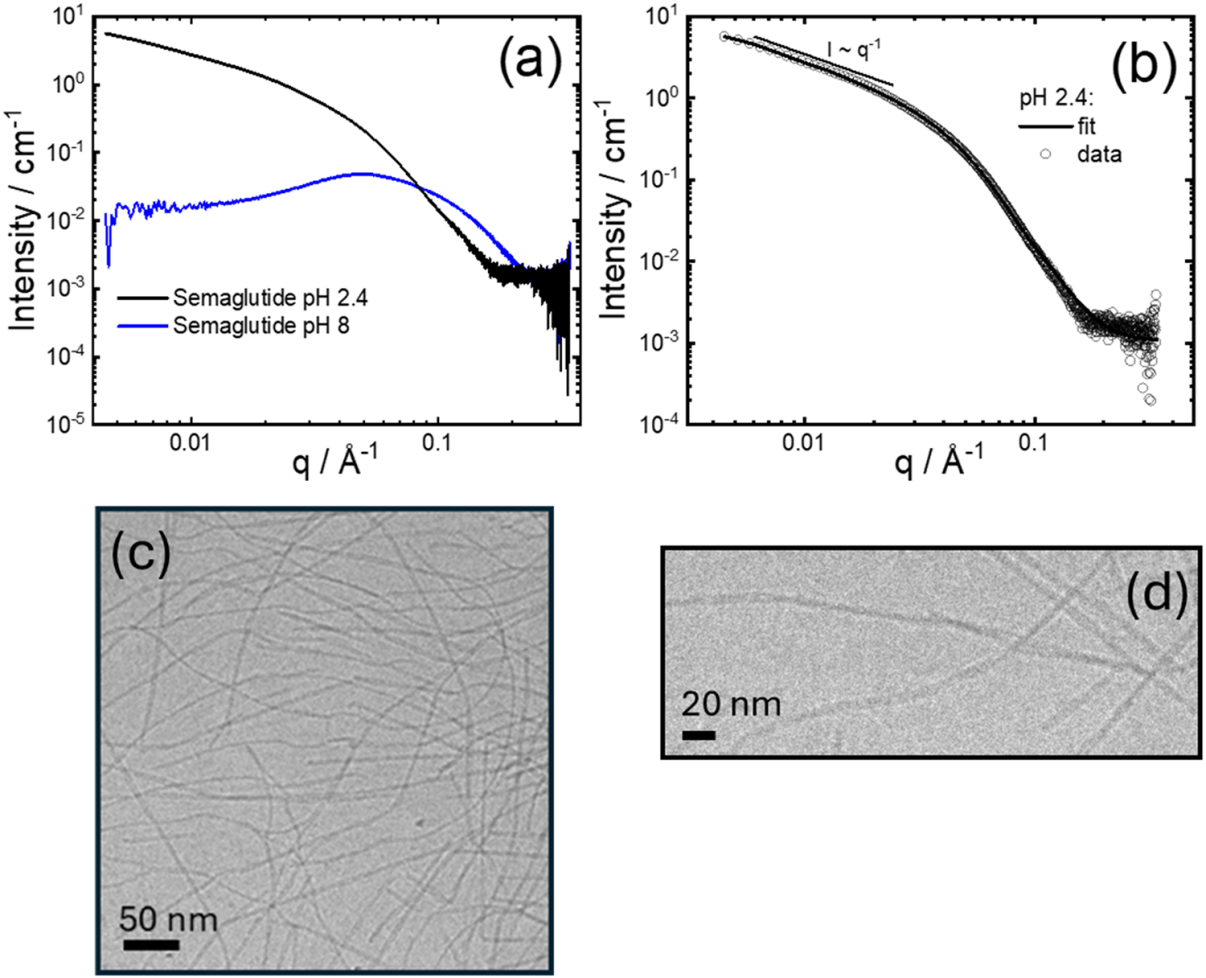
(a) SAXS data for 1 wt % solutions comparing semaglutide (base) pH 8 and semaglutide (TFA) pH 2.4 (aged samples), (b) SAXS data for 1 wt % solution of semaglutide (TFA) pH 2.4. Open symbols: measured data, lines: form factor fit described in the text. For ease of visualization, only every 5th data point is plotted. (c) Cryo-TEM image for 1 wt % solution of semaglutide TFA, pH 2.4. (d) Enlarged cryo-TEM image showing twisted fibrils in a pH 2.4 solution.

The change in the self-assembled structure comparing semaglutide (TFA) at pH 2.4 with semaglutide (base) at pH 8 must be influenced by the charge and its balance (and potentially the nature of the counterion for the TFA salt). The titration curve of a 1 wt % semaglutide (TFA) solution was measured and is shown in Figure S4. A solution at pH 2.4 is below the apparent p*K*_a_ of the acidic residues. Considering the three positively charged residues at this pH (His and two Arg) and the N-terminus, the naïve net charge is estimated to be +4, which is approximate since pH 2.4 is close to the p*K*_a_ of some carboxyl groups, which may further be influenced by the environment and/or aggregation.^41–45^ The fibrils can form through hydrogen bonding with a contribution from electrostatic interactions due to the nonuniform distribution of charged and hydrophobic residues (Scheme 1). This was examined in more detail through MD simulations and is discussed in more detail below. The pH-dependent peptide conformation for semaglutide (TFA) at pH 2.4 was examined by using FTIR and CD spectroscopies. The amide I region of the FTIR spectrum for a fresh sample, shown in Figure 2a, has a peak at 1672 cm^–1^ due to bound TFA counterions^46–48^ and a peak at 1649 cm^–1^ due to α-helical structure.^49,50^ The spectrum was compared to that for a 40-day-aged sample, this aging period being selected based on prior work, which shows the development of aggregation features such as a fluorescence spectrum peak over this time scale for a semaglutide derivative.^25^ Slow aggregation (over days) was noted for GLP-1 itself^17^ and aggregation over weeks was observed for semaglutide in buffer,^26^ and our own prior study on semaglutide micellization at pH 8.^27^ The FTIR spectrum in Figure 2a for an aged sample shows the clear development of a peak at 1622 cm^–1^, which is a signature for the development of β-sheet structure.^49,50^ This behavior can be contrasted with that for semaglutide (base) at pH 8, the amide I FTIR spectra of which are shown in Figure S5. The spectra for both fresh and aged samples show a broad maximum at 1645 cm^–1^, characteristic of a mix of α-helical and disordered structure consistent with our previous CD analysis.^27^ The spectra for fresh and aged samples have the same shape, although there is a change in the absorbance.

**Figure 2:**
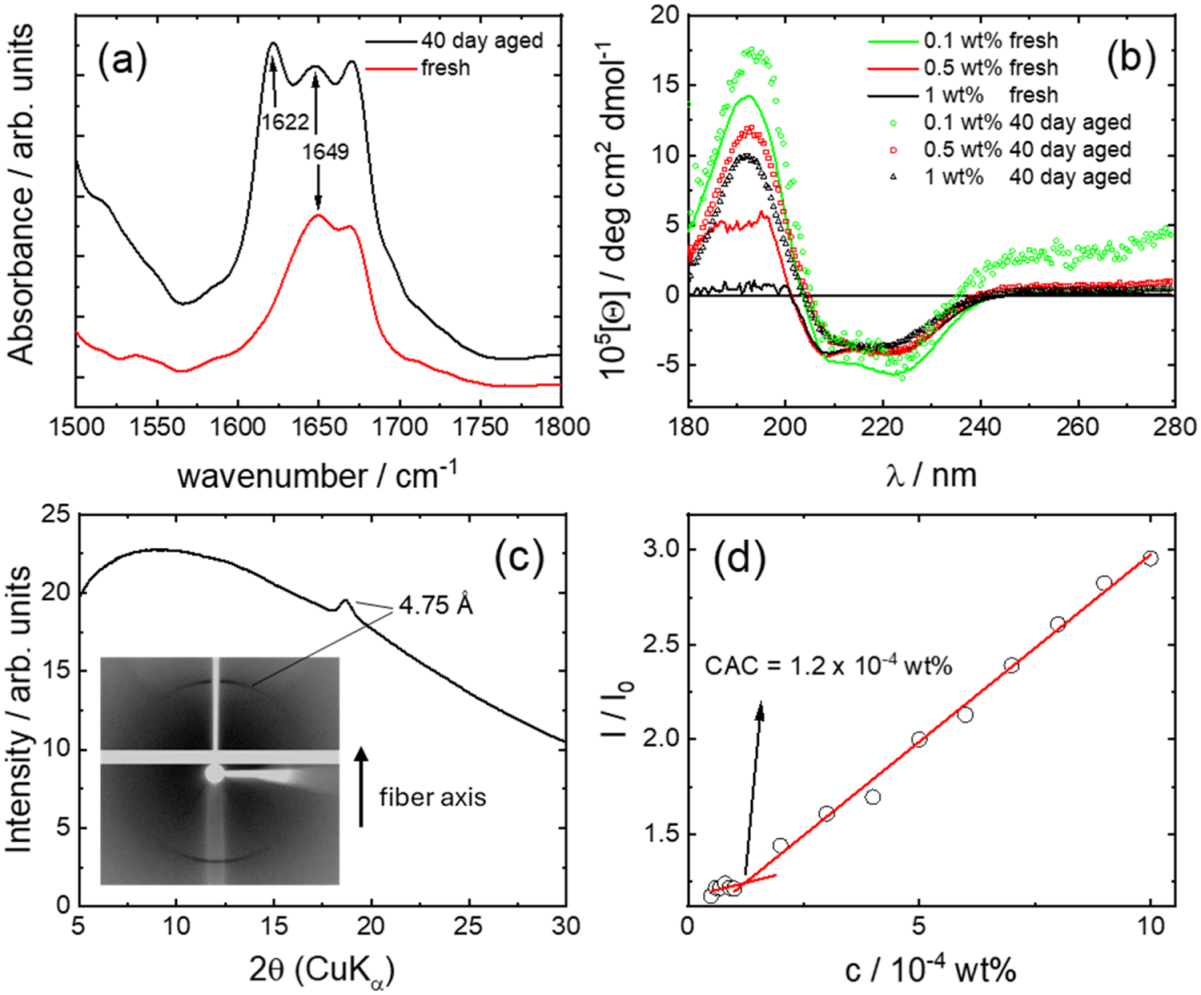
(a) FTIR spectra from 1 wt % solutions for fresh and 40-day-aged samples of semaglutide (TFA) (pH 2.4). (b) CD spectra for semaglutide (TFA) at the concentrations indicated that the pH was measured to be 1 wt % (pH 2.4), 0.5 wt % (pH 2.6), and 0.1 wt % (pH 3.1). (b), (c) Fiber XRD—intensity profile obtained from the 2D image inset, which shows meridional orientation of peaks due to β-strand spacing. (d) Determination of critical aggregation concentration (CAC) using ThT fluorescence (intensity at λ = 486 nm) for a semaglutide (TFA) pH 2.4 sample.

FTIR measurements were complemented with CD spectroscopy. Spectra for a fresh sample of semaglutide (TFA) at pH 2.4, shown in Figure 2b, exhibit the signature of α-helical structure with a double minimum pattern at 208 and 222 nm. The spectra in Figure 2b reveal a significant change: after 40 days of aging, the molar ellipticity is substantially decreased for all concentrations across the wavelength range measured. In addition, the spectra now exhibit the signature of the β-sheet structure, with broad minima near 216 nm and maxima at around 190 nm. The CD spectra therefore support FTIR and show that there is a major conformational shift from α-helix in fresh samples of semaglutide (TFA, pH 2.4) to β-sheet after 40 days of aging. CD spectra were also recorded for intermediate pH values, and the measurements indicate that by decreasing pH starting from pH 8 (semaglutide base native pH), the α-helix structure starts to be lost between pH 8 and 4 (Figure S6). Alternatively, by increasing pH starting from pH 2.4 (semaglutide TFA native pH), the β-sheet signature minimum near 216 nm starts to be lost, and the α-helix component of the spectra develops at pH 6–7 (Figure S7).

The reversibility of the transition from α-helical to β-sheet structures was examined by CD spectroscopy. The spectra in Figures S6 and S7 show a good degree of reversibility considering both starting conditions and pH change routes, i.e., semaglutide base pH 8 down to pH 2.4 and back up to pH 8 (Figure S6) or semaglutide TFA pH 2.4 up to pH 12 and back down to pH 2.4 (Figure S7). This data also indicates that the distinct secondary structures can be accessed at a given pH independent of the semaglutide form (base or TFA salt), i.e., these structures are pH-dependent, although there are differences in the position of the β-sheet minimum, which is red-shifted in the data for semaglutide (base) solutions, which were observed to be cloudy. This CD red-shift^51^ and cloudiness are due to the formation of extended β-sheet fibrils.

The β-sheet structure within an aged pH 2.4 semaglutide (TFA) sample was further confirmed by fiber X-ray diffraction. The data shown in Figures 2c and S8a show a peak at *d* = 4.75 Å, which is a classical signature of β-sheet structure, representing the interstrand spacing.^52,53^ In addition, the meridional orientation of the peaks in the 2D pattern (Figures 2c and S8a) shows that the β-strands are oriented perpendicular to the fibril long axis. This data was further reinforced by *in situ* synchrotron WAXS on a fiber (data shown in Figure S8b), which shows a sharp peak at *d* = 4.76 Å (β-strand spacing) and a broad peak at *d* = 7.9 Å, which may be associated with the β-sheet spacing.

Having established that semaglutide forms β-sheet fibrils in a TFA solution at pH 2.4, the critical aggregation concentration (CAC) was obtained from the concentration dependence of fluorescence intensity of the ‘amyloid’ dye Thioflavin T.^54,55^ The data in Figure 2d show a change in slope of the fluorescence intensity (normalized to that of a ThT solution, *I*_0_) at a CAC = (1.2 ± 0.2) × 10^–4^ wt %, and all aggregation experiments were performed with samples above this concentration. The original fluorescence spectra are shown in Figure S9, which also contains a plot of the fluorescence intensity at higher concentration, which exhibits a change in slope at 0.022 wt %, this being ascribed to the onset of sample turbidity.

The structure of the semaglutide fibrils was modeled by atomistic MD simulations. An extensive range of starting structures was explored, which led to a model shown in Figure 3a that comprises hydrogen-bonded β-strand arrays arranged in an opposed arrangement of two antiparallel β-sheets with a core of the lipidated lysine derivative (Lys-20 substitution termed Lyc) side chains. This led to fibrils in which β-strands curved from an initial linear arrangement during the simulation, along with spontaneous twisting of the β-sheets in the fibrils as the simulation progressed. Movie S1 shows this clearly for a 128-strand fibril comprising two antiparallel sheets. The splaying of the peptide strands is ascribed to the distribution of charged, polar, and hydrophobic residues in the semaglutide sequence (Scheme 1). The preponderance of charged residues at the termini is notable, which leads to fraying at the β-strand termini due to electrostatic repulsion of like-charged residues (especially arginines at the C-terminus). The presence of anionic residues at the N-terminus and cationic ones at the C-terminus may stabilize an opposed antiparallel arrangement, as shown in Figure 3a. In addition, the facial distribution of hydrophobic residues and polar/charged residues may favor interfacial curvature, i.e., preferential segregation of the hydrophobic residues on the interior and hydrophilic ones on the exterior of fibrils comprising splayed β-strands. The twisting of the fibrils observed during MD runs is in agreement with the cryo-TEM images (e.g., Figure 1d). An additional atom-resolved image from the MD simulations is shown in Figure S10. The solvent-accessible surface area (SASA) and related parameters were obtained from the simulation runs and are shown in Figure S11. The SASA reduces as the system reaches equilibrium; however, since the starting state is prebuilt fibrils, an aggregation propensity (AP) based on SASA^56^ is not a suitable parameter to assess the formation of a nanostructure from an initial unordered configuration, whereas for our previous simulations on micelles, an AP value was defined.^27^

**Figure 3:**
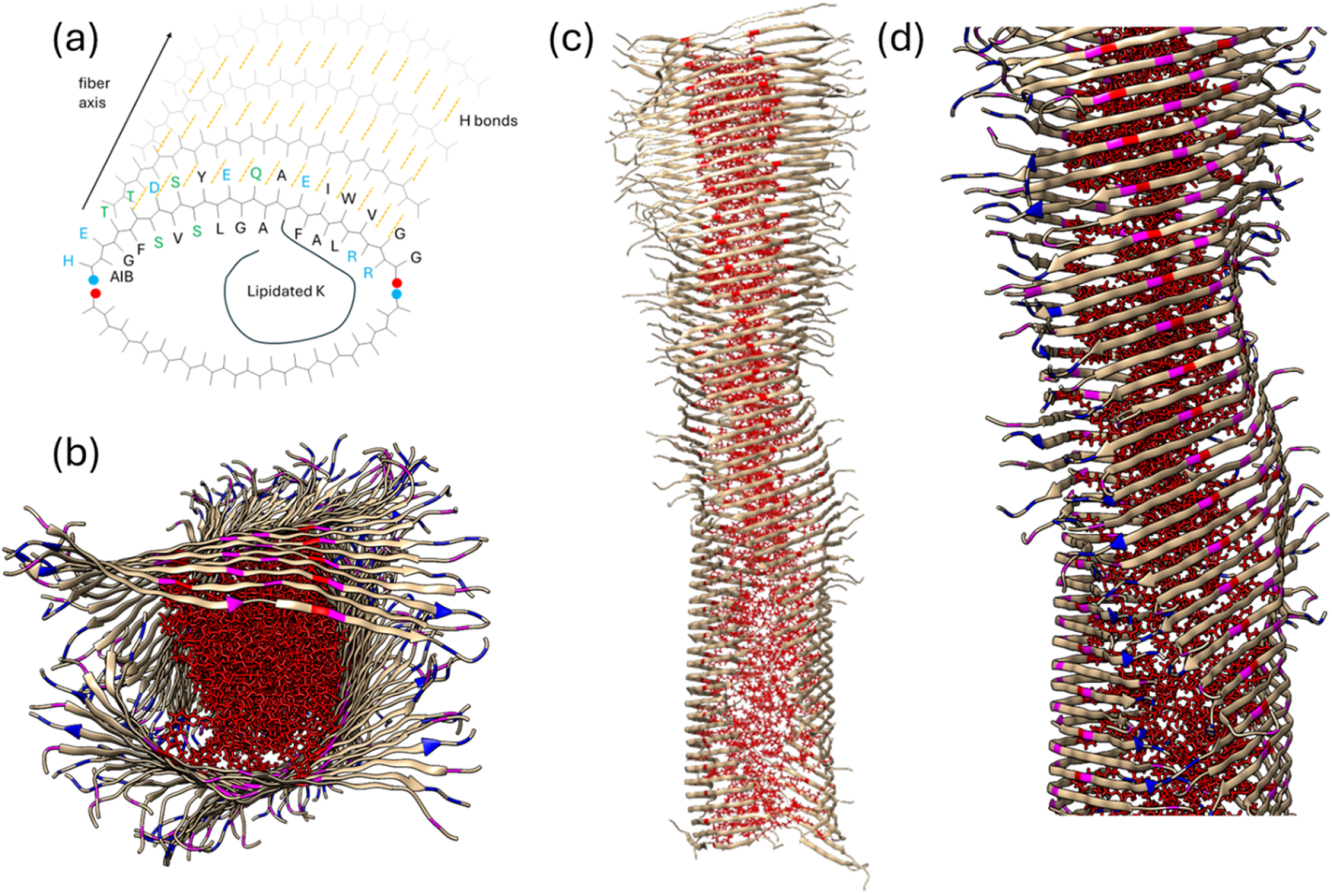
Model for semaglutide fibrils from MD simulations. (a) Schematic showing β-strand with labeled residue color coded as follows: blue, charged; green, polar; black, hydrophobic; red dot, C-terminus; and blue dot, N-terminus. (b) Image along fibril axis showing the Lyc core in red chains, Glu residues in magenta, and Arg residues in blue. Panel (c) showing twisted fibril structure with β-strands and red Lyc interior. Panel (d) shows an enlargement of the twisted fibril structure with selected residues colored as in panel (a).

The modeled fibril structure can be contrasted with the micelle-like clusters previously simulated^27^ for semaglutide based on experimental observations for pH 8 samples. Images of micelles also showing the α-helical conformation of the chains are provided in Figure S12.

The fibril dimensions from the MD simulations are consistent with the SAXS modeling, which yields a total cross-section radius (Table S1) *R* = 45 – 50 Å with core radius 20 Å. Cross-sectional density profiles (orthogonal to the fibril principal axis) were computed from averaged MD simulation frames and are shown in Figure S13; these show that the lipidic (Lyc) core has dimensions of 20 Å, while most of the density lies within a radius of 50 Å of the fibril core. The MD density profiles also show that the Arg residues lie at the exterior, while the most prominent anionic residues (Glu) lie in the main β-sheet density maximum of the outer part (β-sheet region) of the fibril.

Our experiments and MD simulations reveal that semaglutide undergoes pH-dependent self-assembly in aqueous solution, forming micelles in the base solution at pH 8 and β-sheet fibrils for semaglutide (TFA) at pH 2.4. We also unexpectedly found that starting from solutions of the semaglutide (base), adding crotonic acid as a carboxyl group- bearing acid to promote electrostatic interactions and hydrogen-bond formation,^57^ and hence vitrification, it was possible to prepare glasses of semaglutide from the base form as shown in Figure 4. The glasses were prepared by slow evaporation of precursor solutions containing either 8 wt % semaglutide in 0.21% crotonic acid (pH 7) or 10 wt % semaglutide in 0.21% crotonic acid at pH 9. The images in Figure 4a,b show that this process enables molding of the glasses. Figure 4c shows that the glasses are fluorescent under illumination with visible light. A fluorescence spectrum is shown in Figure S14. The fluorescence is ascribed to the presence of a tryptophan residue in the semaglutide sequence (Scheme 1). Remarkably, we found that the α-helical structure observed for semaglutide under native conditions^27^ is retained in the glass, as shown by the CD spectrum in Figure 4d, which contains the characteristic signature^58–60^ of this secondary structure with minima at 209 and 222 nm. We are not aware of prior reports on glasses in which defined peptide secondary structures are vitrified, and this is considered as a type of chiral-imprinted glass. The glass shows high transmittance over an extended wavelength in the UV/visible region, as shown in Figure 4e. At lower wavelengths, the transmittance is affected by the absorption of the tryptophan residue around 250 nm.

**Figure 4:**
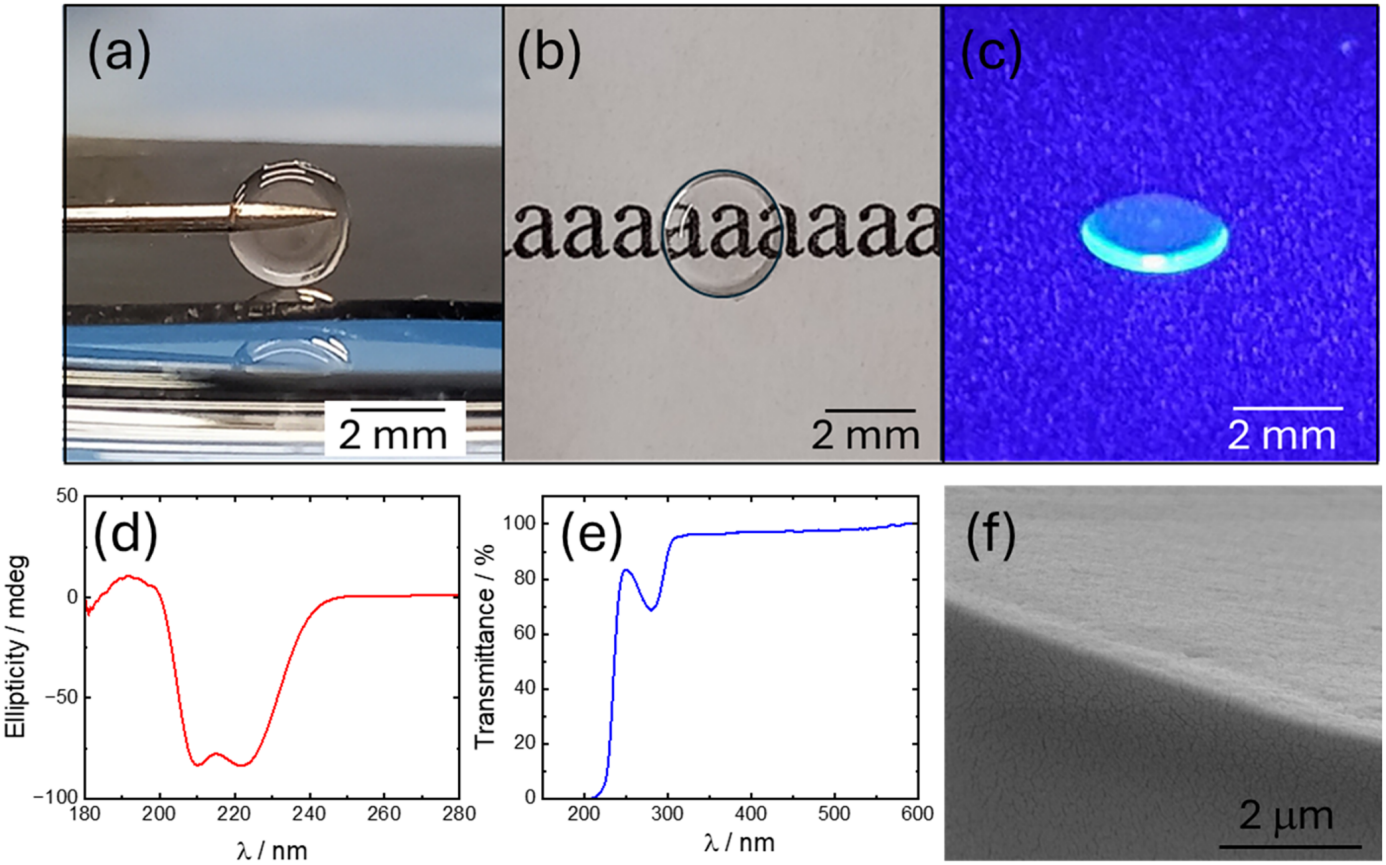
Semaglutide glass prepared from slow evaporated solution of semaglutide (base) in the presence of crotonic acid. (a,b) Images of molded glass. (c) Image showing fluorescence under illumination with λ = 395 nm light. (d) CD spectrum of glass. (e) Transmittance of glass. (f) SEM image showing glass cross-section.

The semaglutide glass is amorphous as shown by SEM, which revealed a featureless morphology (e.g., Figure 4f, additional images in Figure S15). This was further examined by synchrotron SAXS/WAXS data shown in Figure S16, which shows no features above background in the WAXS pattern. The SAXS profile contains a broad peak at *q* = 0.22 Å^–1^. This resembles the broad maximum at *q* = 0.4–0.5 Å^–1^ observed for semaglutide micellar solutions.^27^ It suggests that the glass might correspond to a vitrified network of oligomer or micelle-like structures with increased average spacing (from *d* = 14 Å to *d* = 29 Å), although it may also just be an amorphous structure with a characteristic length scale *d* = 29 Å. Differential scanning calorimetry (DSC) was used to determine the glass transition temperature *T*_g_ = 45.2 °C (Figure S17). This is substantially above room temperature but closer to body temperature, relevant to potential therapeutic uses of the glass.

## Conclusions

In summary, we have demonstrated that semaglutide exhibits a remarkable diversity of aggregation pathways, including pH-dependent self-assembly and evaporation-driven glass formation. At pH 8, oligomers or micelles (depending on concentration) are formed by semaglutide (base), which develops after aging, whereas semaglutide (TFA) at pH 2.4 aggregates into β-sheet fibrils. The pH dependence arises from the presence and distribution of acidic and basic residues. At pH 2.4, electrostatic repulsions between residues are reduced compared to higher pH, and hydrogen-bonded β-sheets are able to form within fibrils, whereas the higher charge at pH 8 leads to repulsion of the peptide C-terminus and the formation of oligomers and micelle-like clusters. This is ascribed to the fact that in the fibrils, an antiparallel arrangement of peptide sequences is possible, while the core lipid chain constraint prevents this in micelles. The charge distribution is also different at pH 8, with a naïve charge of −4 expected under these conditions, based on standard single residue p*K*_a_ values, although as mentioned above, these can be significantly shifted dependent on the local environment and self-assembly.

It is known that proteins and “amyloid” peptides can be “denatured” to form β-sheet fibrillar structures by reduction of pH in acidic conditions.^53,61–64^ Here, we show a similar phenomenon for semaglutide, which is unexpected given that this molecule was designed to be nonaggregating.^8^ In fact, it shows pH-dependent aggregation behavior, self-assembling into oligomers/micelles at pH 8 and fibrils at pH 2.4. The former is closest to conditions relevant to its formulation for application. The acidic pH 2.4 is lower than that in tissues such as the pancreas, brain, or gastrointestinal tract, which are among sites where GLP-1R-expressing cells are present.^65–67^ However, our CD data (Figure S6) point to the presence of at least some β-sheet structures at physiologically relevant pH values. Open questions for future research include the important issue of the potential relationship between bioactivity and the aggregation state. In addition, it will be interesting to further investigate the influence of counterions on the pH-dependent transition between oligomers/micelles and fibrils, and the associated potential effects on the fibril structure and the mechanisms and kinetics of the transition.

We present a model based on atomistic molecular dynamics simulations for the semaglutide fibril structure. This is based on consideration of the peptide sequence, which shows a biased distribution of charged, polar, and hydrophobic residues. There is a preponderance of charged residues at the termini, hydrophilic (charged and polar) residues on one face, and hydrophobic residues on the other. This leads to curvature of the β-strands and fraying of charged residues at the termini due to electrostatic repulsion, while the presence of multiple charged residues at the termini may promote an opposed arrangement of the β-sheets due to salt bridge interactions. The model shows features in excellent agreement with the SAXS data, in particular the dimensions of the core and exterior parts of the fibril, and it is consistent with the hydrogen bonding direction perpendicular to the fibril axis, a constraint from fiber XRD measurements, and the spontaneous twisting agrees with cryo-TEM images of fibrils.

The ability to vitrify semaglutide is unexpected, and the glass has the notable property that it can trap the α-helical structure of the peptide within a vitrified amorphous matrix. We propose that the semaglutide glass may be a vitrified network of oligomers/micelles formed in the precursor solution; at least SAXS indicates a characteristic domain size in the glass. Glasses are considered nonequilibrium states, and the interplay between different equilibrium aggregation pathways and nonequilibrium vitrification observed here is intriguing. These structures may form in preference to the crystallization of the lipopeptide, which has not been reported, and indeed in general lipopeptides are not readily crystallized due to the presence of the lipid chains, which hinders the formation of ordered crystal structure of the attached peptide (further likely to be restricted by the presence of diethylene glycol in the linker chain in semaglutide, Scheme 1). The glass has high transparency and is fluorescent. Further research into related properties, such as circularly polarized luminescence, is planned and is a more detailed examination of material properties. It would also be interesting to examine the use of the semaglutide glass for applications, such as subcutaneous slow-release depots,^68^ for which it might further be desirable to tune the *T*_g_ value by adjusting the starting salt solution (type of salt or concentration) and/or by blending with other glass-formers.

## Supplementary Material





## Notes

HPLC and ESI-MS data, additional cryo-TEM images, additional SAXS data, additional MD data, CD spectra, fluorescence spectra, additional SEM images, SAXS/WAXS data, DSC data, and Table of SAXS fitting parameters (DOCX)
MD simulation trajectory showing curvature of the β-sheet structure in a 128-residue fibril (MPG)
