# Supplementary material for: Fibrillar and Micellar Aggregation of Semaglutide and Formation of a Chiral-Imprinted Glass
Source: Biomacromolecules. 2026 Mar 31;27(4):2818–27. doi: 10.1021/acs.biomac.5c02669 (PMC13080979; doi:10.1021/acs.biomac.5c02669)
Supplement: Supplementary file 1 [file bm5c02669_si_001.docx]

**Supporting Information**

**Fibrillar and Micellar Aggregation of Semaglutide and Formation of a Chiral-Imprinted Glass**

Valeria Castelletto,^1^ Lucas R. de Mello, ^1^ Jani Seitsonen,^2^ Ian W. Hamley,^1,*^

*^1^ School of Chemistry, Food Biosciences and Pharmacy, University of Reading, Whiteknights, Reading, Berkshire, RG6 6AD, U.K.*

*^2^ Nanomicroscopy Center, Aalto University, Puumiehenkuja 2, FIN-02150 Espoo, Finland*

* Author for correspondence: I.W.Hamley@reading.ac.uk

(a)


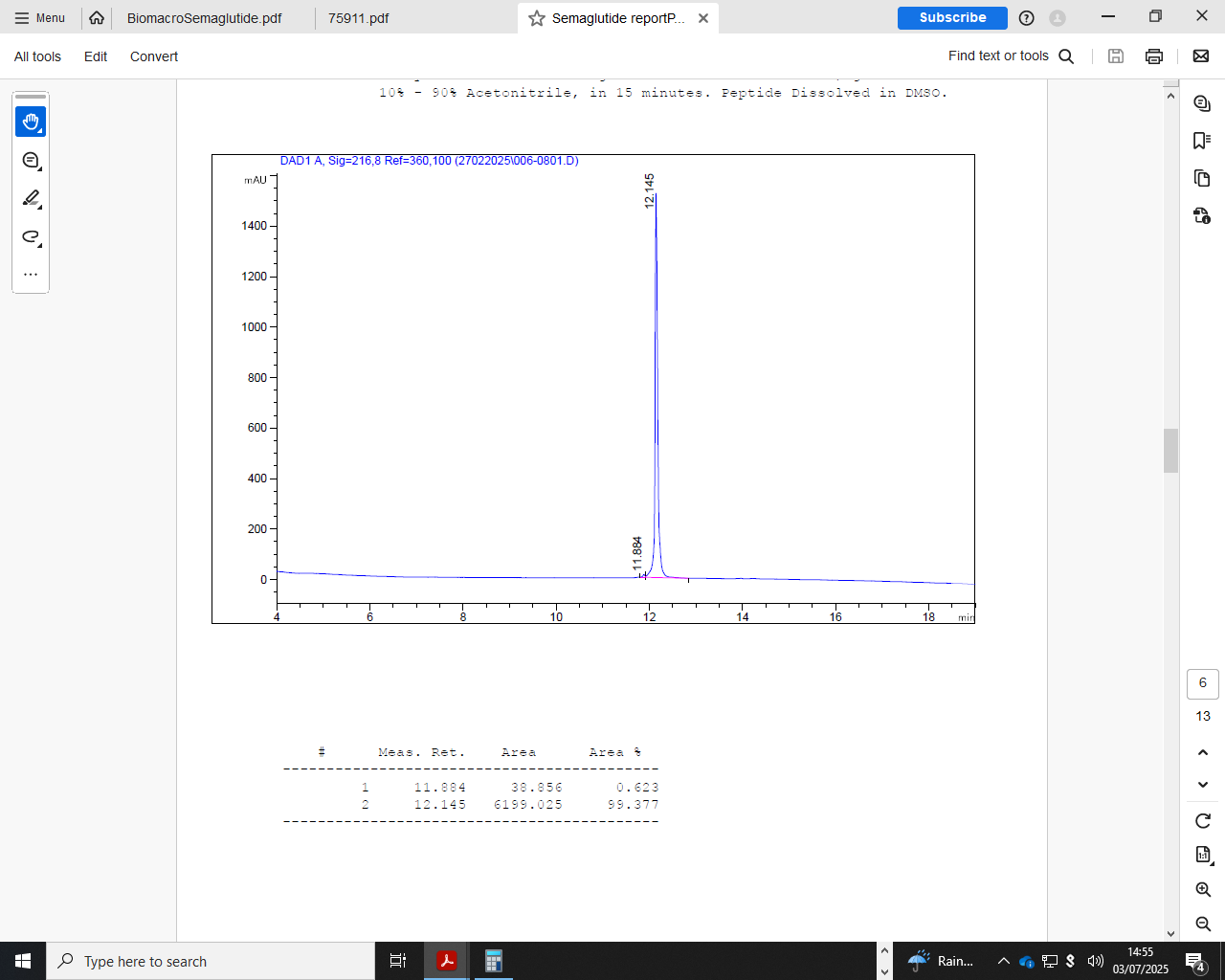


(b)


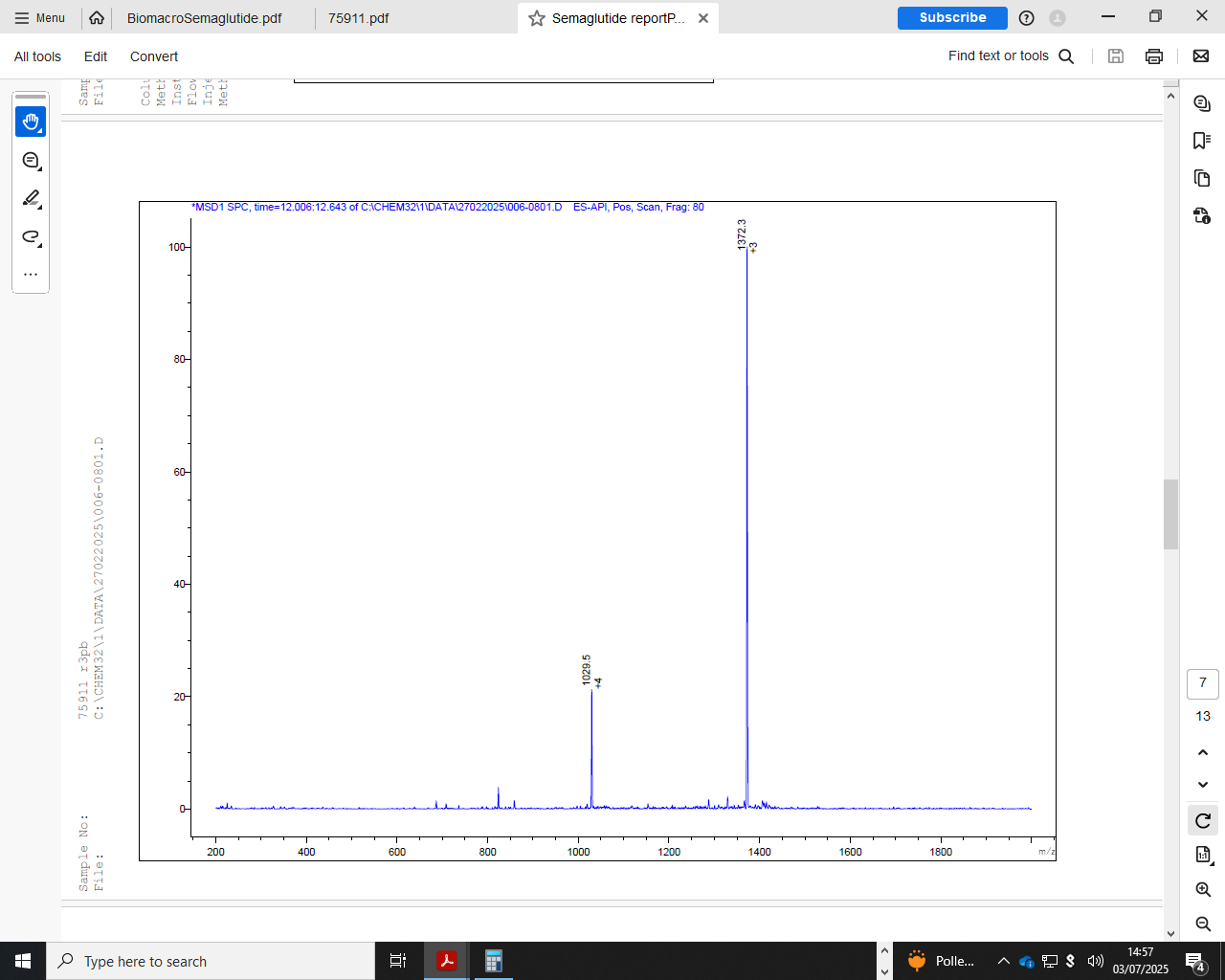


**SI Fig.S1**. Characterization data for semaglutide (TFA). (a) HPLC with peak analysis, (b) ESI-MS.

(a)


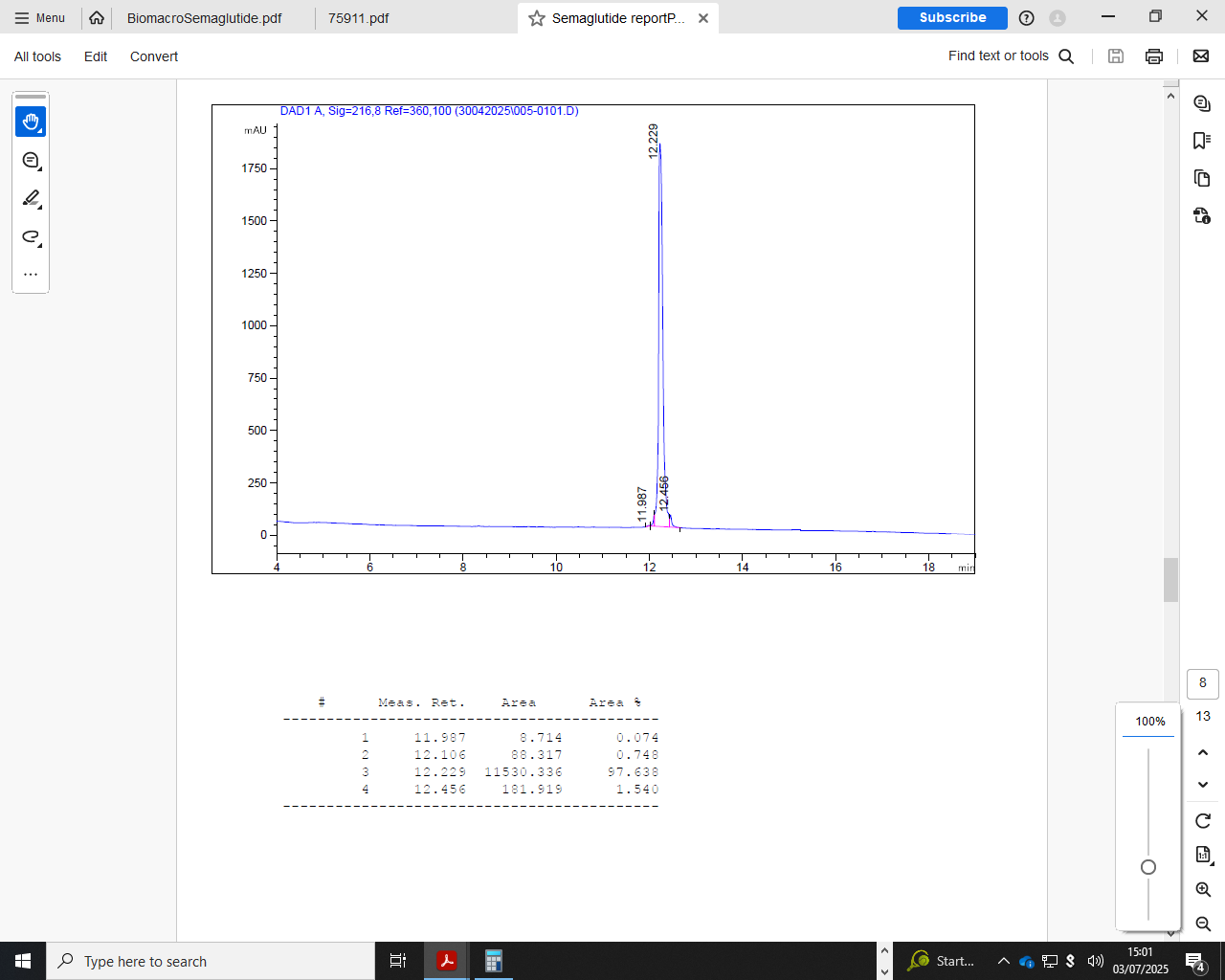


(b)


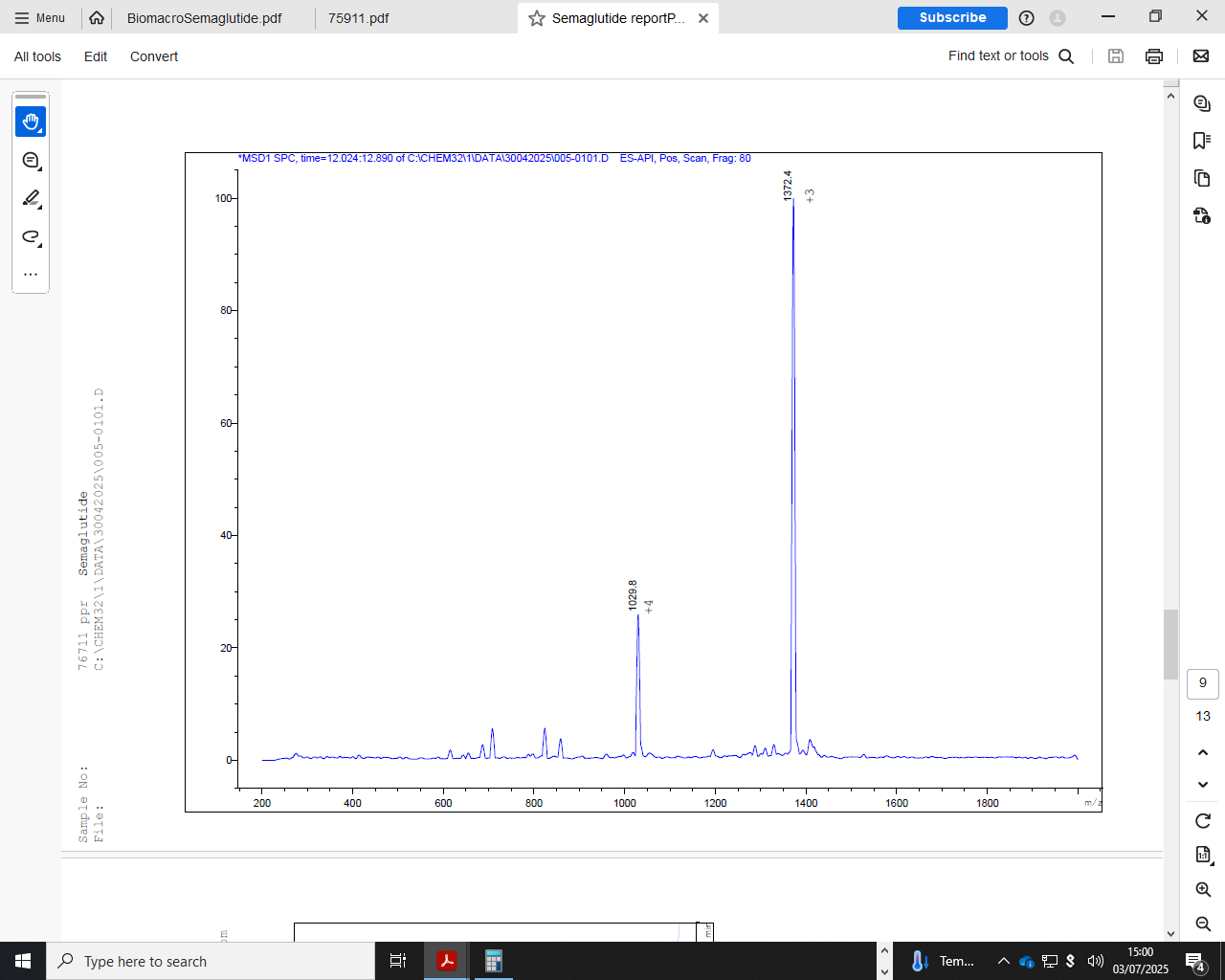


**SI Fig.S2**. Characterization data for semaglutide (base). (a) HPLC with peak analysis, (b) ESI-MS.

(a)





(b)





(c)





(d)


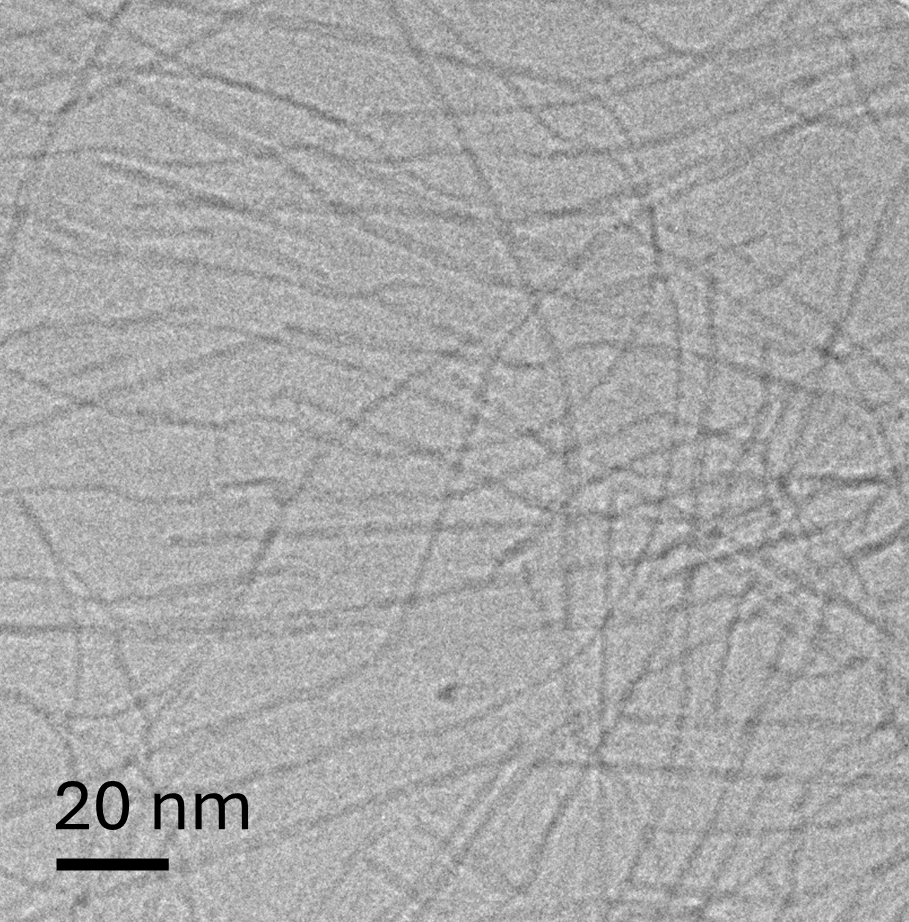


**SI Fig.S3.** (a-d) Examples of additional cryo-TEM images from a 1 wt% solution of semaglutide (TFA) pH 2.4.

**SI Fig.S4.** Titration curve for 1 wt% semaglutide. The three pKa values marked correspond to pKa(1) = 3.2 corresponding to the acidic residues and COOH termini, pKa(2) = 6.6 corresponding to His and the N-terminus and pKa(3) = 12.3 corresponding to the Arg residues.

**SI Fig.S5.** FTIR spectra from 1 wt% solutions for fresh and 40 day aged samples of semaglutide (base) pH 8.

**SI Fig.S6.** CD spectra for 1 wt% solutions of semaglutide (base) at pH values indicated, prepared by adding HCl to an initial native pH 8.4 sample to decrease the pH to 2.4 and then (after 3 months fridge storage) addition of NaOH to increase back to pH 8. The sample is transparent at pH 8 and cloudy at pH 2.4.

**SI Fig.S7.** CD spectra for 1 wt% solutions of semaglutide (TFA) at pH values indicated, prepared by adding NaOH to an initial native pH 2.4 sample up to pH 12 and then (after 3 months fridge storage) addition of HCl to decrease back to pH 2.

(a)


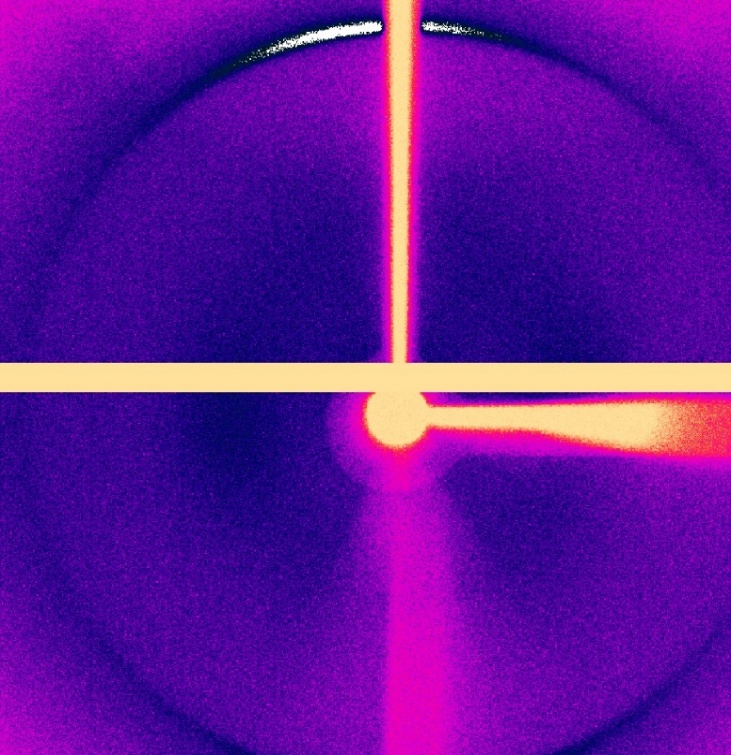


(b)

**SI Fig.8**. X-ray diffraction data for a fiber prepared from an aged pH 2.4 semaglutide (TFA) sample. (a) XRD pattern with longer sample-detector distance (140 mm) showing the meridional reflection at top. (b) In situ synchrotron WAXS profile of a fiber.

**SI Fig.S9**. Thioflavin T fluorescence data used to obtain CAC (Fig.2d). (a) Fluorescence spectra for the semaglutide (TFA) pH 2.4 solutions at the concentrations indicated, (b) Plot of the intensity (normalized to that of the reference ThT solution, I_0_) at higher concentration showing a further discontinuity in gradient.


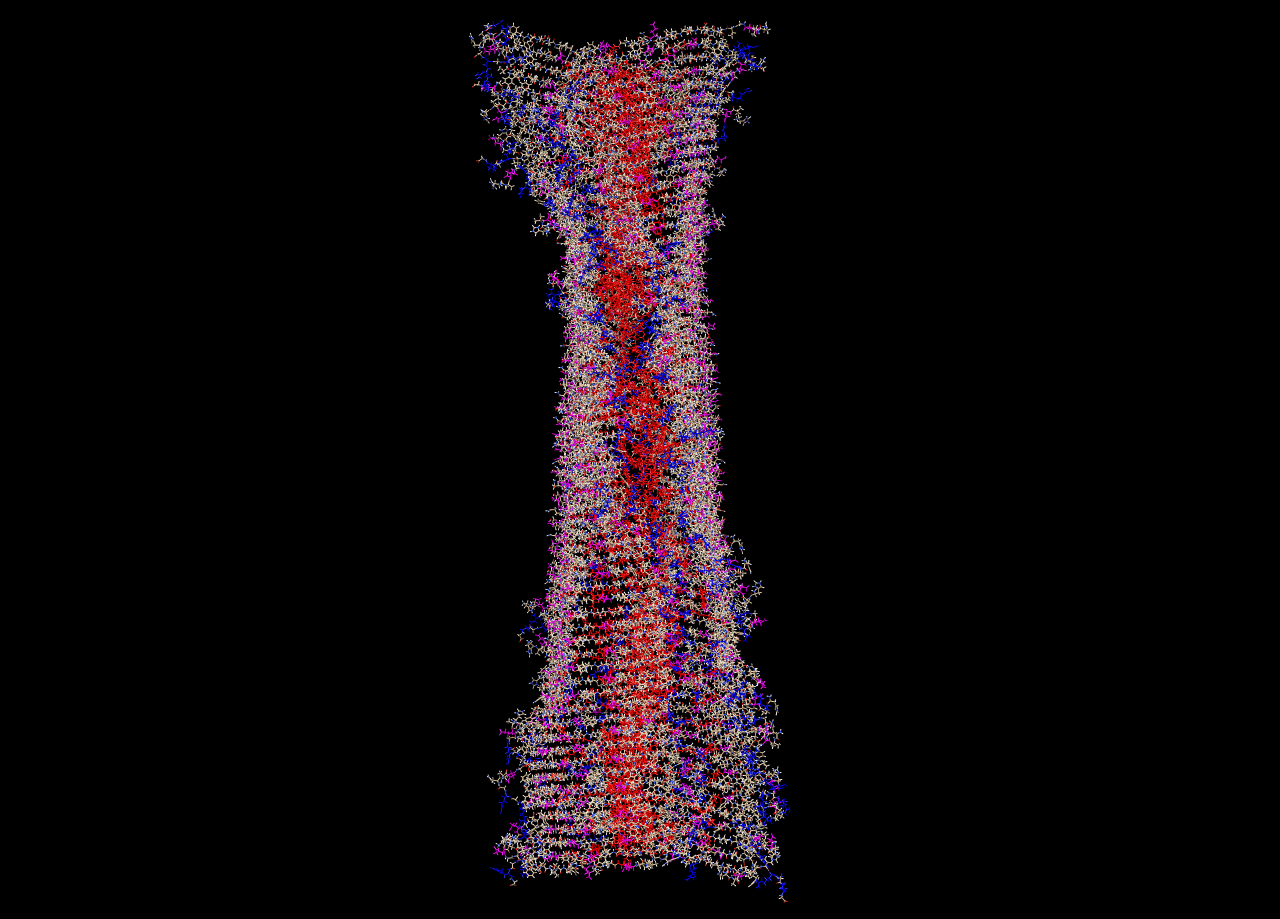


**SI Fig.S10**. All-atom image from MD simulation of a twisted semaglutide fibril with selected residues colored: red – Lyc (lipidated Lys-20), blue – Arg residues, magenta – Glu residues.

**SI Fig.S11**. Solvent-accessible surface area related parameters from MD simulations. Traces for repeat runs of different simulation lengths are shown.


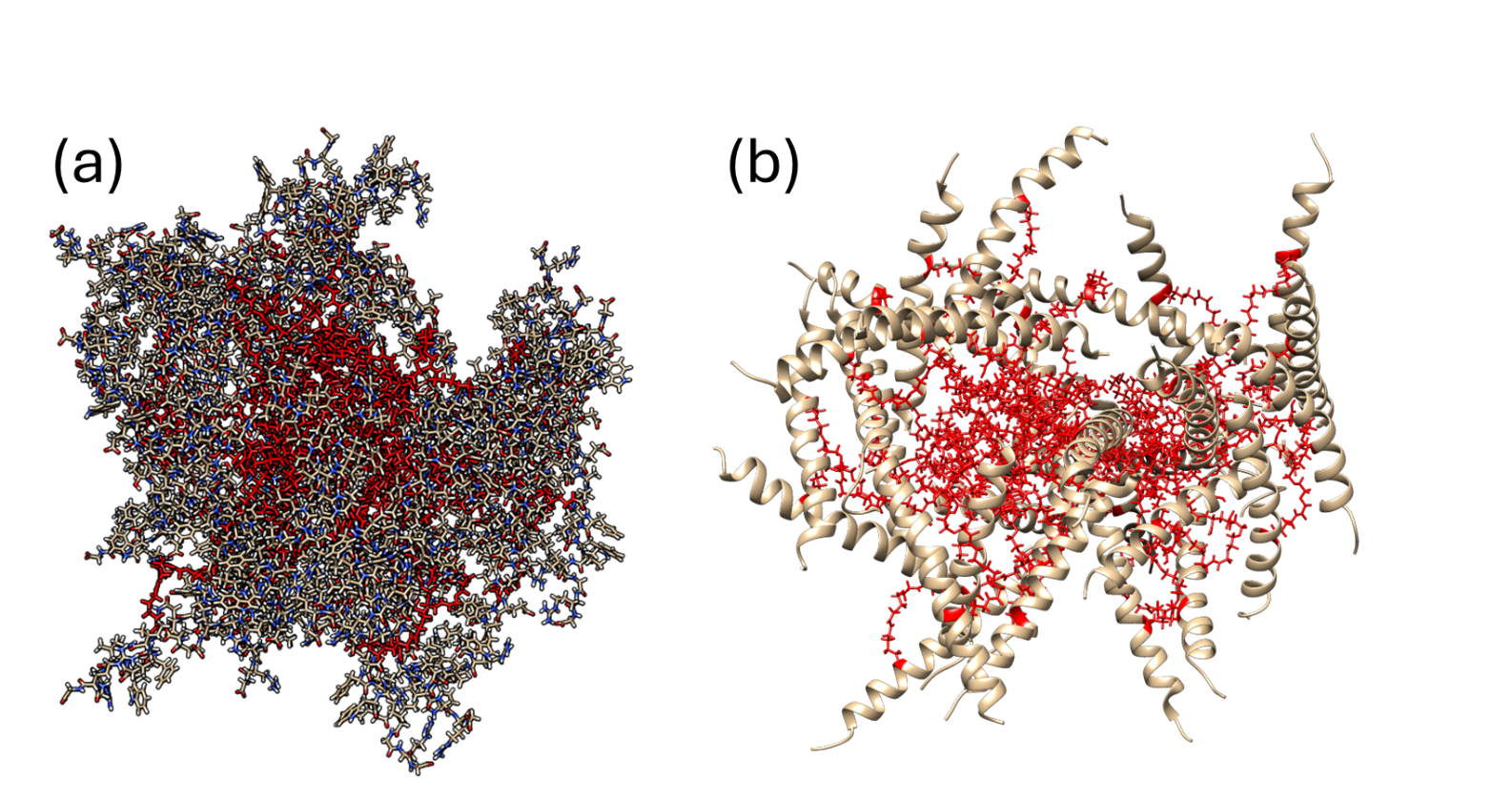


**SI Fig.S12**. Images from MD simulations (final frame) reported previously^1^ for semaglutide micelles redrawn with Lyc colored red. (a) All atom representation, (b) Ribbon representation.

**SI Fig.S13**. Cross-section residue (residue atom count per unit area) density profiles from MD simulation (average over last 20 frames).

**SI Fig.S14**. Fluorescence spectrum from semaglutide glass with λ_ex_ = 280 nm.


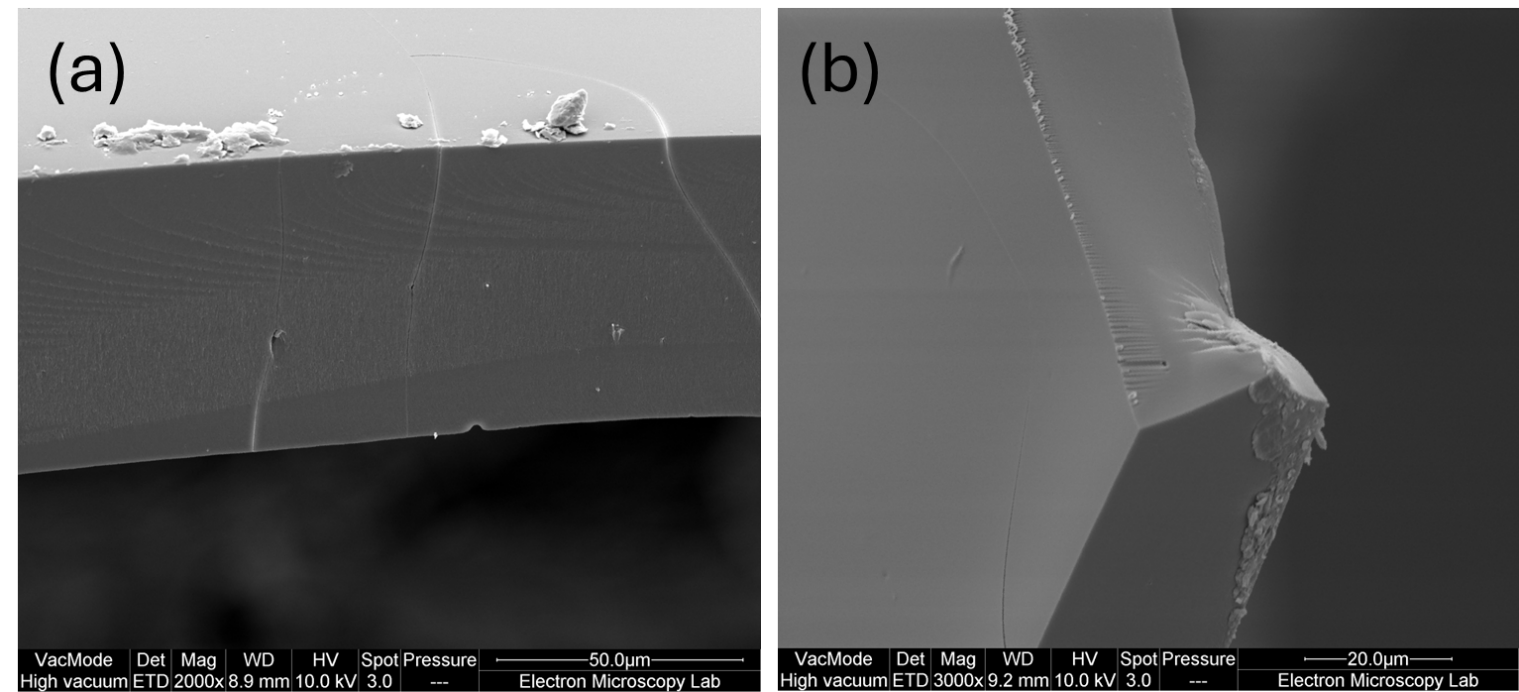


**SI Fig.S15**. Additional cryo-SEM images of semaglutide crotonate glasses.

**SI Fig.S16**. Synchrotron SAXS/WAXS data for a semaglutide crotonate glass sample.

**SI Fig.S17.** DSC data showing glass transition temperature (T_g_) for semaglutide glass.

**SI Table S1**. Fit parameters to the SAXS data in Fig.1b using a long cylindrical shell form form factor. Fitting performed using SASfit.^2-3^

|  | **1 wt%** |
| --- | --- |
| *R*_c_  ± σ_c_ / Å | 20.0 ± 16.5 |
| *t* / Å | 27.1 |
| *L* / Å | 791 |
| *η*_c_ / cm^-1^ | -1.17×10^-6^ |
| *η*_s_ / cm^-1^ | 7.00×10^-8^ |
| BG | 0.001 |

**Key:** **Long Cylindrical Shell** **Form factor**: *R:* cylinder radius (*σ*_c_: Gaussian polydispersity in *R*), *t*: shell thickness, *L*: length, *η*_c_: scattering contrast of core, *η*_s_: scattering contrast of shell **Background**: BG (constant).

**References**

(1) Hamley, I. W.; de Mello, L. R.; Castelletto, V.; Zinn, T.; Cowieson, N.; Seitsonen, J.; Bizien, T., Semaglutide Aggregates into Oligomeric Micelles and Short Fibrils in Aqueous Solution *Biomacromolecules* **2025,** *26*, 3786–3794.

(2) Bressler, I.; Kohlbrecher, J.; Thünemann, A. F., SASfit: a tool for small-angle scattering data analysis using a library of analytical expressions. *J. Appl. Cryst.* **2015,** *48*, 1587–1598.

(3) Kohlbrecher, J.; Bressler, I., Updates in SASfit for fitting analytical expressions and numerical models to small-angle scattering patterns. *J. Appl. Cryst.* **2022,** *55*, 1677–1688.
